# Metagenomic sequencing identified microbial species in the rumen and cecum microbiome responsible for niacin treatment and related to intramuscular fat content in finishing cattle

**DOI:** 10.3389/fmicb.2024.1334068

**Published:** 2024-03-11

**Authors:** Zhuqing Yang, Xiao Chen, Mingjin Yu, Ruixue Jing, Linbin Bao, Xianghui Zhao, Ke Pan, Bihui Chao, Mingren Qu

**Affiliations:** ^1^College of Animal Science and Technology, Jiangxi Agricultural University, Nanchang, China; ^2^Jiangxi Provincial Key Laboratory for Animal Nutrition/Engineering Research Center of Feed Development, Jiangxi Agricultural University, Nanchang, China; ^3^Animal Husbandry and Veterinary Bureau of Guangchang County, Fuzhou, China

**Keywords:** niacin, microbiome, rumen, cecum, intramuscular fat content, castrated finishing steers

## Abstract

**Introduction:**

Niacin is one of the essential vitamins for mammals. It plays important roles in maintaining rumen microecological homeostasis. Our previous study indicated that dietary niacin significantly elevated intramuscular fat content (IMF) in castrated finishing steers. Whether niacin affects fat deposition by regulating the microbial composition and functional capacities of gastrointestinal microbiome has been unknown yet.

**Methods:**

In this study, 16 castrated Xiangzhong Black cattle were randomly assigned into either control group fed with a basal concentrate diet (*n* = 8) or niacin group fed with a basal concentrate diet added 1000 mg/kg niacin (*n* = 8). Seven rumen samples and five cecum content samples were randomly collected from each of control and niacin groups for metagenomic sequencing analysis.

**Results:**

A total of 2,981,786 non-redundant microbial genes were obtained from all tested samples. Based on this, the phylogenetic compositions of the rumen and cecum microbiome were characterized. We found that bacteria dominated the rumen and cecum microbiome. *Prevotella ruminicola* and *Ruminococcus flavefaciens* were the most abundant bacterial species in the rumen microbiome, while *Clostridiales bacterium* and *Eubacterium rectale* were predominant bacterial species in the cecum microbiome. Rumen microbiome had significantly higher abundances of GHs, GTs, and PLs, while cecum microbiome was enriched by CBMs and AAs. We found a significant effect of dietary niacin on rumen microbiome, but not on cecum microbiome. Dietary niacin up-regulated the abundances of bacterial species producing lactic acid and butyrate, fermenting lactic acid, and participating in lipid hydrolysis, and degradation and assimilation of nitrogen-containing compounds, but down-regulated the abundances of several pathogens and bacterial species involved in the metabolism of proteins and peptides, and methane emissions. From the correlation analysis, we suggested that niacin improved nutrient digestion and absorption, but reduced energy loss, and Valine, leucine and isoleucine degradation of rumen microbiome, which resulted in the increased host IMF.

**Conclusion:**

The results suggested that dietary manipulation, such as the supplementation of niacin, should be regarded as the effective and convenient way to improve IMF of castrated finishing steers by regulating rumen microbiome.

## Highlights

- The phylogenetic compositions and potential functional capacities were profiled and compared between rumen and cecum microbiomes in castrated finishing steers.
- Dietary niacin up-regulated the abundances of rumen bacterial species producing lactic acid and butyrate, fermenting lactic acid, and participating in lipid hydrolysis, but down-regulated the abundances of several pathogens and bacterial species involved in the metabolism of proteins and peptides, and methane emissions.
- Dietary niacin increased host intramuscular fat content by regulating rumen microbiome.
- The supplementation of niacin should be regarded as the effective and convenient way to improve cattle IMF by regulating rumen microbiome.

## 1 Introduction

Niacin (Vitamin B3) is one of the essential vitamins for mammals. It is widely used as a feed additive in livestock production because of its crucial roles in numerous catabolic and anabolic redox processes, such as lipid metabolism, tissue oxidation, glycolysis, and respiratory functions (Chilliard, 1993) by serving as the precursor of NAD+/NADH and NADP+/NADPH (Flachowsky, 1993). In addition to being a coenzyme precursor, niacin has been known for its antilipolytic effect by activating the hydroxycarboxylic acid 2 receptor (HCA2), which effectively reduces the levels of all atherogenic lipoproteins including VLDL and LDL subclasses as well as Lp (a), and increases the levels of the protective lipoprotein of HDL (Carlson, 2005; Pescara et al., 2010). Recent studies have demonstrated that the pharmacological dose of niacin did provide beneficial effects on animal health, particularly in high-yielding dairy cows under stress conditions (Kristensen and Harmon, 2004; Pescara et al., 2010; Titgemeyer et al., 2011; Luo et al., 2017, 2019; Zou et al., 2022), likely attributing to niacin’s positive influence on lipid metabolism or vasodilation (Carlson, 2005; Pescara et al., 2010). Additionally, niacin plays important roles in converting ruminant volatile fatty acids (VFAs), such as acetate, into long-chain fatty acids (Chilliard, 1993). This may promote the fatty acid synthesis and result in increased intramuscular fat (IMF) deposition that further impacts muscle tenderness, juiciness, and meat color (Yang et al., 2016).

The complex microbial community effectively ensures rumen fermentation in ruminants, which primarily converts feedstuffs into essential nutrients, such as VFAs, ammoniate, vitamins, and microbial proteins (Doreau and Ottou, 1996; Kristensen and Harmon, 2004; Zou et al., 2022). Niacin has been proven to promote rumen microbial growth, maintain rumen microecological homeostasis, and avoid lactate accumulation in the rumen wall (Doreau and Ottou, 1996; Kristensen and Harmon, 2004; Luo et al., 2017). Supplementation of 800 mg/kg niacin in a high-concentrate diet could stabilize ruminal pH and alleviate subacute rumen acidosis (SARA) by reducing the abundance of *Streptococcus bovis* or by improving NAD^+^ concentration to inhibit the activity of lactate dehydrogenase (Kristensen and Harmon, 2004; Luo et al., 2017). Dietary niacin also inhibited starch utilization and stimulated fiber degradation primarily by reducing the relative abundance of *Proteobacteria*, *Succiniclasticum*, *Acetivibrio*, and *Treponema*, or increasing the abundance of *Prevotella* (Kristensen and Harmon, 2004). A recent study has demonstrated that niacin supplementation might alleviate heat stress by promoting the proliferation of bacteria belonging to phylum *Succiniclasticum*, which in turn contributed to the cellulose digestion and metabolic function improvement of beef cattle under heat-stress conditions (Zou et al., 2022). Dietary niacin should also enhance the number and density of rumen protozoa (Doreau and Ottou, 1996). Niacin was useful for avoiding ketogenic problem on rumen fermentation in dairy cows (Yuan et al., 2012). It should also optimize rumen fermentation for improving nutrient digestibility and increasing cattle productivity through influencing the rumen microbiota, metabolic processes, and the ability to enhance feed intake in bulls (Flachowsky, 1993; Luo et al., 2017, 2019). However, few studies have focused on the effects of niacin on the composition and functional capacities of rumen and cecum microbiota by metagenomic sequencing analysis, and the relationship of the niacin-induced changes in rumen and cecum microbiome with host fat deposition in beef cattle has been largely unknown.

Our previous study found that dietary niacin significantly elevated IMF content, marbling score, and the activities of NADH-related enzymes (G6PDH and ICDH) in muscle. Furthermore, it also increased serum HDL-C concentration and decreased the levels of serum LDL-C, triglyceride, non-esterified fatty acid, total cholesterol, and glycated serum protein (Yang et al., 2016). Based on this, we hypothesized that niacin should modulate lipid and glucose metabolisms by regulating the microbial composition and functional capacities of gastrointestinal microbiome in beef cattle. Therefore, in this study, we investigated the effect of dietary niacin on the composition and functional capacities of rumen and cecum microbiome in experimental cattle, identified microbial taxa affected by dietary NA, and evaluated the correlations of niacin-induced changes in rumen and cecum microbiome with host muscle IMF.

## 2 Materials and methods

### 2.1 Animal management and sample collection

The detailed information about experimental cattle, and their feeding and managements were described in our previous study (Yang et al., 2016). In brief, a total of 16 Xiangzhong Black cattle with similar body weight (575.50 ± 31.50 kg) at the same age of 24 months were used in this study. All 16 Xiangzhong Black cattle were castrated steers and were randomly assigned into either the control group fed with a basal concentrate diet (*n* = 8) or the niacin group fed with a basal concentrate diet added 1,000 mg/kg niacin (*n* = 8). The components and their proportions of the basal concentrate diet are shown in Supplementary Table S1. The experimental cattle were individually housed in pens and provided *ad libitum* with a diet consisting of 90% concentrate and 10% forage straw. After a 120-day formal experiment, all cattle were humanely slaughtered following the Chinese standard procedures at a commercial abattoir (Hunan, China). Luminal content was collected from multiple regions of each experimental cattle’s rumen within 30 min after slaughter. After mixed well, rumen sample was put into a 7-ml sterile centrifuge tube. Cecum luminal content was also harvested from the middle part of each experimental cattle’s cecum within 30 min after slaughter. The samples were immediately immersed in liquid nitrogen and subsequently transferred to a −80°C refrigerator. All experimental cattle were healthy and did not receive any probiotic or antibiotic treatment throughout the whole feeding experiment (from 1 month before the feeding experiment to sampling). All processes related to experimental animals were conducted in accordance with the guidelines established by the Ministry of Agriculture and rural affair of China. The Animal Care and Use Committee (ACUC) of Jiangxi Agricultural University approved this study (No. JXAU2011-006).

### 2.2 Measurements of cattle fat deposition traits

The measurements of cattle fat deposition traits including backfat thickness, IMF content, and marbling score for all 16 experimental Xiangzhong Black cattle were described in our previous study (Yang et al., 2016). In brief, the backfat thickness was measured from the left side of the carcass using a vernier caliper. The mean value obtained from triplicate measurements was treated as the phenotypic value of each animal. Longissimus dorsi (LD) muscle samples were dissected from the 12–13th ribs of the right carcass to assess the marbling score and measure IMF content. Marbling score was determined at 24 h postmortem following the Japan marbling grading standards (8–12: abundance, 5–7: moderate, 3–4: mean, 2: a little, and 1: trace) by an experienced panelist. IMF content of LD muscle was measured by diethyl ether extraction using a Soxhlet apparatus. Furthermore, eye muscle area (EMA), which reflects meat production in cattle, was also phenotyped using graph paper method.

### 2.3 Microbial DNA extraction of rumen and cecum content samples, library construction, and metagenomic sequencing

Microbial DNA was extracted from rumen and cecum content samples using the QIAamp Fast DNA Stool Mini Kit (Qiagen, Germany) following the manufacturer’s protocols. The concentration and the quality of DNA samples were evaluated using a NanoDrop-1000 (Thermofisher Scientific, United States) and 0.8% agarose gel electrophoresis. DNA libraries for metagenomic sequencing were constructed following the manufacturer’s instructions (Illumina, United States). The clustering of the index-coded samples was performed on a cBot Cluster Generation System according to the manufacturer’s instructions. After cluster generation, the libraries were sequenced on a Novaseq 6000 platform (Illumina, United States) adopting a 150-bp paired-end sequencing strategy with insertion size of 350 bp.

### 2.4 Bioinformatic analysis of metagenomic sequencing data

Adaptor sequences and low-quality sequence reads were removed from the raw sequence data with fastp (v0.19.4) (Chen et al., 2018). And then, the remaining reads were further mapped to the bovine reference genome (ARS-UCD1.2) to remove the contamination of host DNA sequences by BWA MEM (v0.7.17-r1188) (Li and Durbin, 2009). Clean sequence reads of each sample were assembled into contigs individually by MEGAHIT (v1.1.3) (Li et al., 2016). Prodigal (v2.6.3) was used to predict genes of assembled contigs (Hyatt et al., 2010). Complete genes containing both start codon and stop codon were retained for further analyses. A non-redundant gene catalog was constructed by clustering all predicted genes at the 90% identity level of protein sequences following the UniRef guidelines (Suzek et al., 2015) by CD-HIT (v4.8.1) (Li and Godzik, 2006). The protein sequences of all non-redundant genes were then aligned to the Uniprot TrEMBL database by DIAMOND (v2.0.8) (Buchfink et al., 2015) at the threshold of e-values ≤1e−5. The taxonomic classification of non-redundant genes was determined based on the lowest common ancestor algorithms by BASTA (v1.3) (Kahlke et al., 2018) at the thresholds of identity >80%, an alignment length >25, and shared by at least 60% of hits.

Functional pathway classification of predicted non-redundant genes was performed by aligning genes to the KEGG (Kyoto Encyclopedia of Genes and Genomes) with the DIAMOND (v2.0.8). The KOBAS (v3.0) was used to retrieve KO annotation results (Xie et al., 2011). Carbohydrate-active enzymes (CAZymes) were annotated by aligning genes to dbCAN database (HMMdb V8) (Huang et al., 2018) with hmmscan program in HMMER (v3.1b2) (Pattabiraman and Warnow, 2021). For all functional annotations, the annotated hit(s) with the highest-score was used for the subsequent analyses (Li et al., 2014, 2018; Backhed et al., 2015).

To estimate the abundance of microbial genes, FeatureCounts (v1.6.2) (Liao et al., 2014) was used to compute the number of successfully assigned clean reads to the gene catalog in each sample. The gene abundances were normalized to fragments per kilobase of gene sequence per million reads mapped (FPKM) (Munk et al., 2018). The abundances of microbial taxa, KEGG pathways, and CAZymes were calculated by adding the abundances of all the members belonging to each category.

### 2.5 Statistical analysis

The α-diversity of rumen and cecum microbial compositions including Observed species, Shannon index, Simpson and Chao 1 index, and the principal coordinate analysis (β-diversity) based on the Bray-Curtis distance were calculated by *vegan* in R package (v4.1.2). Wilcoxon rank-sum test was used to compare the α- and β-diversity of rumen and cecum microbiome between niacin and control groups. Results were visualized by ggplot2 package. The linear discriminate analysis effect size (LefSe) algorithm (Segata et al., 2011) was used to identify microbial taxa, KEGG pathways, and CAZymes showing significantly different abundances between niacin and control groups at the significance threshold of *p* < 0.05 and |LDA| score > 2.0. The results were visualized with the boxplots or heatmaps plotted by *ggpubr* and *pheatmap* in R package (v4.1.2).

The correlations between niacin-regulated rumen bacterial species and fat deposition traits, between niacin-regulated KEGG pathways and fat deposition traits, and between niacin-regulated rumen bacterial species and KEGG pathways were evaluated by Spearman correlation analysis. Benjamin Hochberg was used to correct the false discover rate (FDR). FDR < 0.2 and *p* < 0.05 was set as the significance level and *p* < 0.1 was considered as the tendency to significant correlation. The results were visualized with the ggplot2 and ComplexHeatmap packages in R software (V.4.1.2).

## 3 Results

### 3.1 Metagenomic sequencing data of rumen and cecum content samples from experimental beef cattle

Seven rumen and five cecum content samples were randomly collected from eight castrated bulls in each of niacin-treated group and control group. Finally, a total of 14 rumen and 10 cecum content samples were used in this study. High throughput metagenomic sequencing of microbial DNA samples generated 13.68 Gigabase pairs (Gb) of raw sequence data per sample (ranging from 10.21 to 18.31 Gb). After filtering the adaptors and low-quality sequences, a total of 327.20 Gb of high-quality clean sequence data were obtained from these 24 samples with an average sequencing depth of 13.63 Gb/sample, ranging from 10.18 to 18.24 Gb (Supplementary Table S2).

*De novo* assembly generated 5,448,212 contigs with the length >1,000 bp. These contigs had the mean length of 2226.36 bp and the maximum length of 495,287 bp. More than 94.49% of contigs showed the length of 1.0 ~ 5.0 kb (Supplementary Table S3). The N50 and N90 values were 2,331 bp and 1,135 bp, respectively. Gene prediction identified 14,591,586 microbial genes from all 24 samples. These genes were clustered at the 90% identity of amino acid sequences following the model of UniRef (Suzek et al., 2015). A microbial gene catalog containing 2,981,786 non-redundant microbial genes were obtained with the average gene length of 831.14 bp and the maximum length of 37,287 bp (Table 1). Among 2,981,786 non-redundant genes, 2,055,110 genes were commonly identified in both rumen and cecum content samples, 567,240 genes were unique to the microbiome of rumen samples, and 359,436 genes were specifically identified in cecum content samples (Supplementary Figure S1A). Most of these 2,981,786 non-redundant genes showed low abundances in tested samples. There were only 840,693 genes with abundance > average abundance and 2,032,104 genes having abundance < average abundance (Supplementary Figure S1B).

**Table 1:** The summary results of contig assembly and non-redundant gene catalog.

	Item	Number
Contig	Contig number	5,448,212
	N50 (bp)	2,331
	N90 (bp)	1,135
	Max length of contigs (bp)	495,287
	Mean length (bp)	2226.36
Gene	Genes number	2,981,786
	N50 (bp)	1,074
	Max length (bp)	37,287
	Mean length (bp)	831.14
	KEGG orthologs/ortholog number	562,385 (18.86%)/3,779
	KEGG pathways	413
	CAZy families	581,777 (19.51%)

### 3.2 Characterization of the phylogenetic compositions of the rumen and cecum microbiome in experimental beef cattle

To determine the phylogenetic compositions of the rumen and cecum microbiome, taxonomic annotation was performed by blasting 2,981,786 non-redundant microbial genes to the Uniprot TrEMBL (known proteins). At the phylum level, a total of 36 phyla were detected in rumen samples, including 24 bacterial phyla, one archaeal phylum, nine Eukaryota phyla, and two viral phyla. The bacterial phyla occupied more than 96.0% of relative abundance. Bacteroidetes (45.93% ± 5.609%, mean ± SD), Firmicutes (42.441% ± 6.192%), Fibrobacteres (5.966% ± 4.254%), and Proteobacteria (1.429% ± 0.277%) were the predominant bacterial phyla in the cattle rumen microbiome. Euryarchaeota was the only archaeal phylum detected in this study with a relative abundance of 0.160% ± 0.100%. The relative abundances of two Protozoa phyla of Ciliophora (0.145% ± 0.073%) and Apicomplexa (0.06% ± 0.032%) were also listed in the top 10 in tested rumen samples (Supplementary Table S4; Supplementary Figure S2A). Similarly, we identified 35 phyla in cecum content samples. Except Candidatus Gracilibacteria, all phyla identified in rumen samples were also observed in cecum content samples, including one archaeal phylum, nine Eukaryota phyla, and two viral phyla. Compared to rumen samples, cecum samples had a higher relative abundance of bacteria (99.6% vs. 96.0%). Firmicutes (73.246% ± 4.841%, mean ± SD), Bacteroidetes (22.663% ± 4.822%), Proteobacteria (2.709% ± 1.337%), and Spirochaetes (0.753% ± 0.759%) were the predominant bacterial phyla in the cecum microbiome. Except Euryarchaeota (Archaea, 0.195% ± 0.086%), no other non-bacterial phyla were listed in the top 10 in relative abundances in the cecum microbiome (Supplementary Table S4; Supplementary Figure S2B).

**Figure 1:**
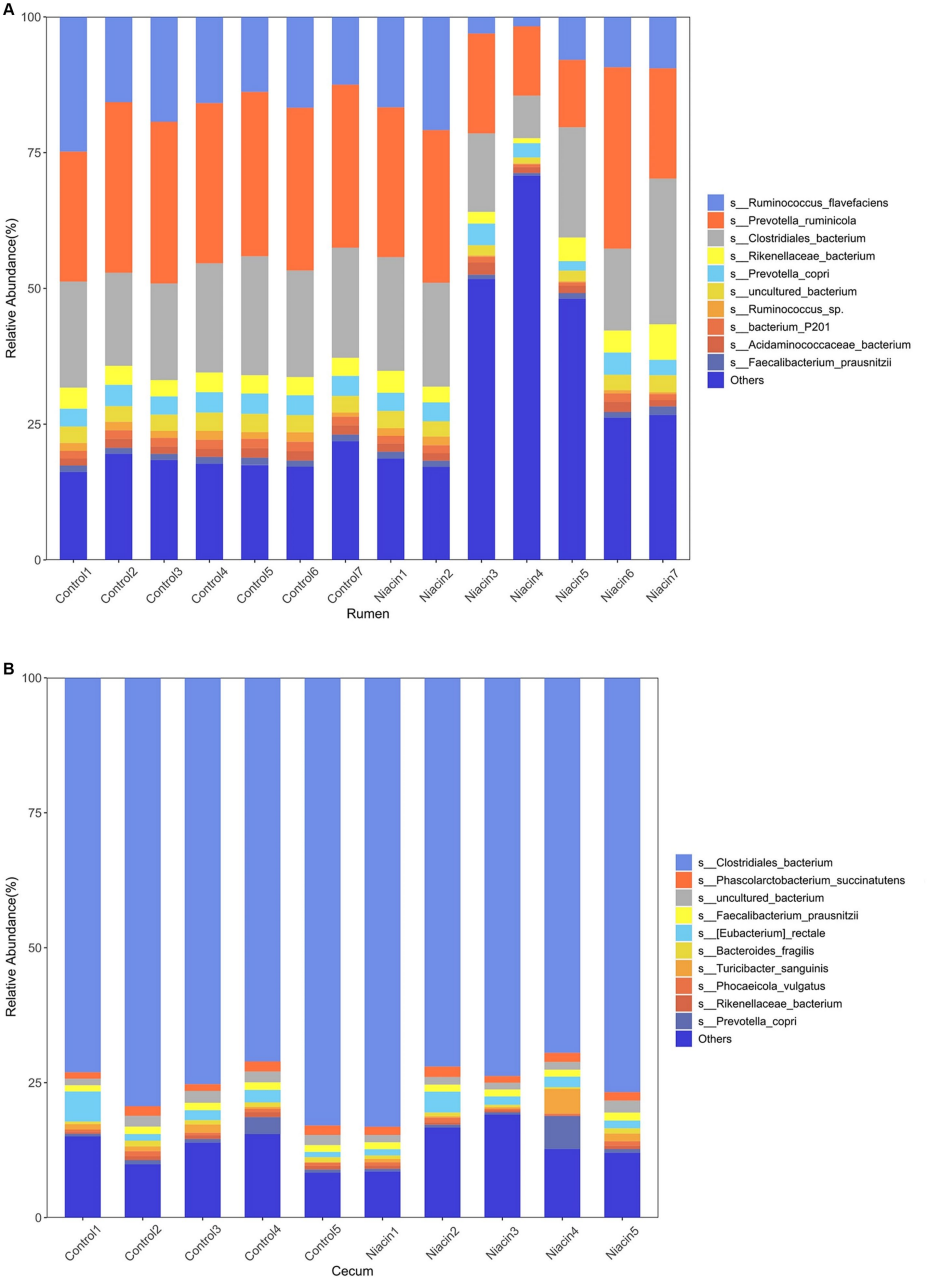
Microbial compositions of rumen and cecum microbiome in tested samples. **(A)** Histogram showing microbial composition of rumen samples at the species level. **(B)** Histogram showing microbial composition of cecum content samples at the species level.

At the species level, a total of 316 microbial species were identified in rumen samples, including 302 bacterial species, four archaeal species, and 10 eukaryota species. Among them, 214 species were present in all 14 rumen samples (Supplementary Table S5). The relative abundances of *Prevotella ruminicola* (25.56% ± 6.902%), *Clostridiales bacterium* (18.643% ± 4.318), and *Ruminococcus flavefaciens* (13.408% ± 6.567%) were listed in the top three (Figure 1A). In cecum content samples, we identified 304 microbial species including 294 bacterial species, four archaeal species, and six eukaryota species (Supplementary Table S6). All the species whose abundances were ranked in the top 10 belonged to Bacteria. *Clostridiales bacterium* (75.686% ± 4.799%) had the highest abundance, followed by *Eubacterium rectale* (2.190% ± 1.453%) and *Phascolarctobacterium succinatutens* (1.583% ± 0.274%) (Figure 1B). It was worthy to note that all four archaeal species including *Methanobrevibacter smithii*, *Methanobrevibacter millerae*, *Methanobrevibacter olleyae*, and *Methanobrevibacter oralis* identified in both rumen and cecum content samples belonged to methanogens. There were 25 species specifically identified in rumen samples, including three eukaryota species, five *Fibrobacter* spp., three *Prevotella* spp. and two *Butyrivibrio* spp. that can produce VFAs. We also identified 13 species that specifically existed in cecum content samples. All 13 species belonged to bacteria (Supplementary Table S6).

### 3.3 Comparison of functional capacity profiles between rumen and cecum microbiome

**Figure 2:**
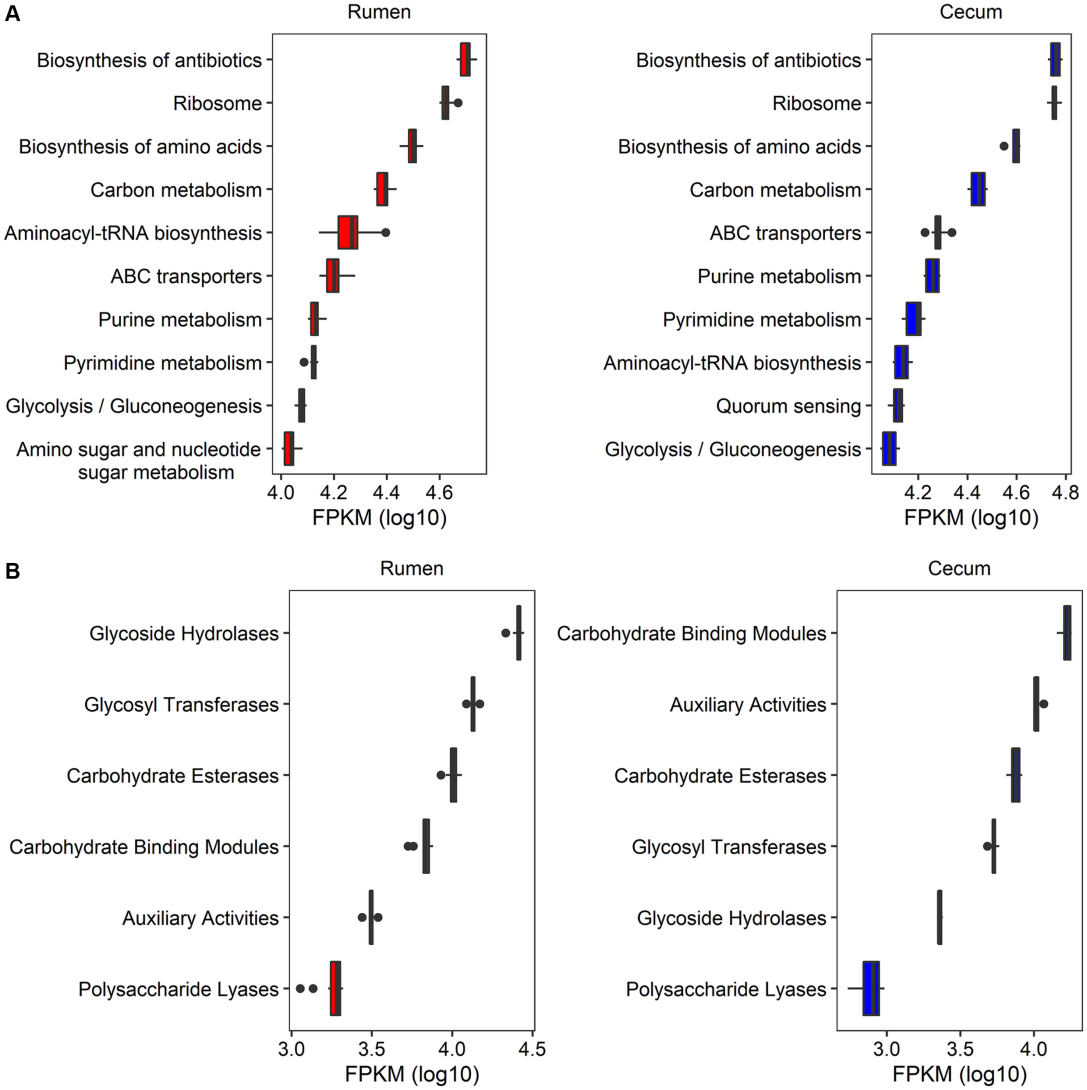
The profiles of functional capacities of rumen and cecum microbiome. **(A)** The top 10 KEGG pathways in abundances in rumen and cecum microbiome. **(B)** The abundances of CAZymes in rumen and cecum microbiome. The *x*-axis shows the log_10_ transformed abundance (FPKM) of function items, and the *y*-axis indicates the items of functional capacities.

Functional capacity profiles of rumen and cecum microbiome were determined by blasting 2,981,786 non-redundant microbial genes to the databases of KEGG and CAZyme. A total of 562,385 (18.86%) of non-redundant genes could be annotated to 3,779 KEGG orthologous groups (KOs) and 413 KEGG pathways (Table 1). The predominant KEGG pathways and their abundances were similar between the rumen and cecum microbiome. The top 10 KEGG pathways in relative abundance in both gastrointestinal tract locations included Biosynthesis of antibiotics, Ribosome, Biosynthesis of amino acids, Carbon metabolism, ABC transporters, Aminoacyl-tRNA biosynthesis, Pyrimidine metabolism, Purine metabolism, and Glycolysis/Gluconeogenesis (Figure 2A; Supplementary Table S7).

There were 581,777 (19.51%) genes that could be annotated to CAZyme categories (Table 1). The categories Carbohydrate Binding Modules (CBMs), Auxiliary Activities (AAs), Carbohydrate Esterases (CEs), Glycosyl Transferases (GTs), Glycoside Hydrolases (GHs), and Polysaccharide Lyases (PLs) were identified in both rumen and cecum microbiome, but their abundances were significantly different in two gastrointestinal sites. Rumen microbiome had significantly higher abundances of GHs (11.2 folds), GTs (2.5 folds), and PLs (2.3 folds) than cecum microbiome. However, cecum microbiome was enriched by CBMs (2.5 folds compared to rumen microbiome) and AAs (3.3 folds) (Figure 2B; Supplementary Table S8).

### 3.4 Identification of microbial taxa and potential functional capacities responding to the niacin treatment

**Figure 3:**
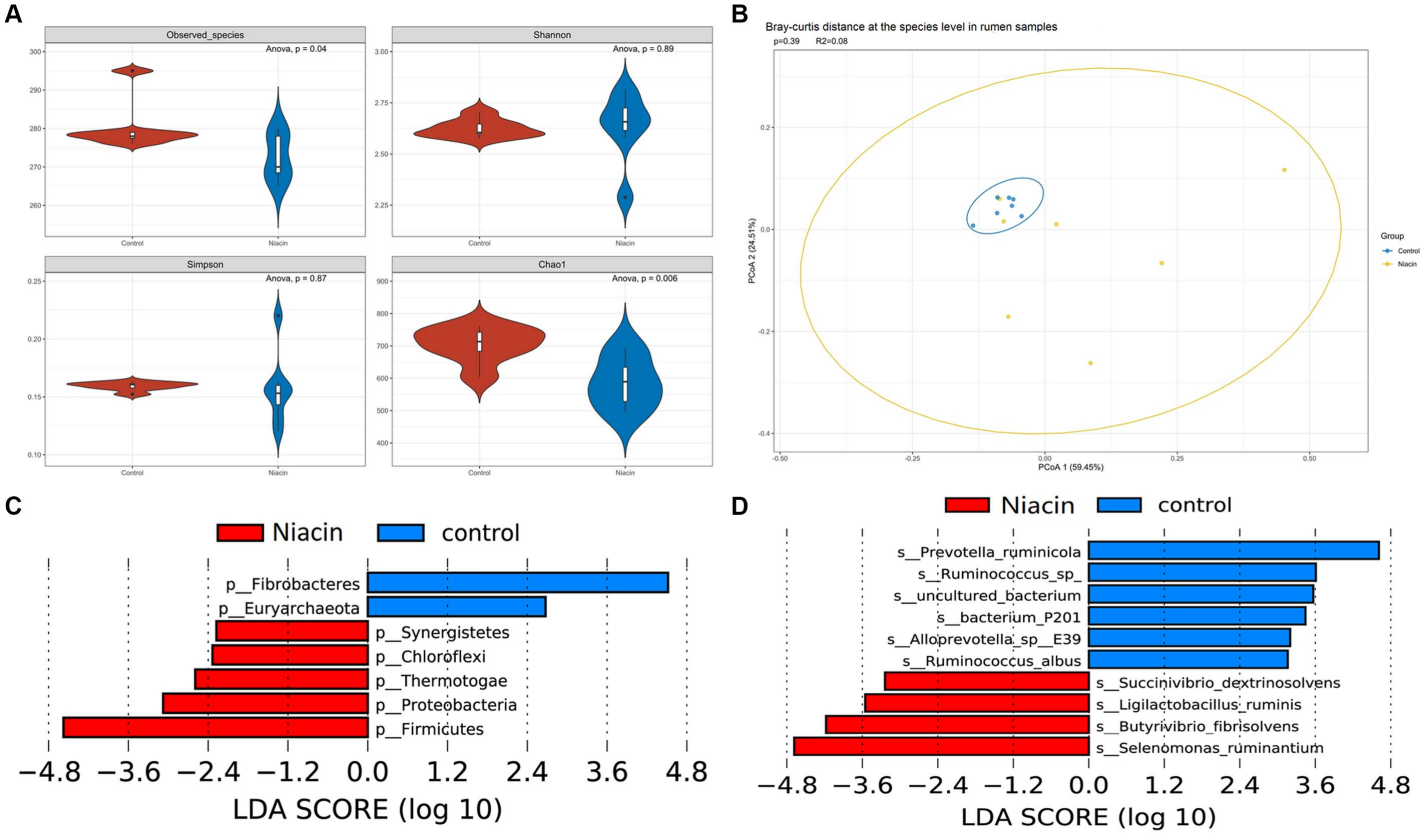
The effect of dietary niacin on rumen microbial composition at the species level. **(A)** The effect of dietary niacin on the α-diversity of rumen microbiome at the species level. The indices of observed species, Shannon, Simpson, and Chao 1 were compared between control and niacin groups using ANOVA analysis. *p* < 0.05 was treated as the significance threshold. **(B)** The effect of dietary niacin on the β-diversity of rumen microbiome. The β-diversity was evaluated based on the bray-curtis distance. **(C)** Differential microbial phyla identified by LefSe analysis between Niacin and control groups. **(D)** Microbial species showing different abundances between Niacin and control groups identified by LefSe analysis. The histogram only shows the differential microbial species with LDA ≥ 3 and *p* < 0.05. All differential microbial species identified are provided in Supplementary Table S9.

The 16 experimental cattle were randomly divided into two groups that were treated with 1,000 mg/kg niacin (*n* = 8, Niacin group) and 0 mg/kg niacin (*n* = 8, control group), respectively. Among 14 rumen samples used in this study, seven samples were randomly collected from niacin group and the other seven samples randomly from control group. Five cecum content samples were randomly collected from each of niacin and control groups. This provided us an opportunity to investigate the effect of dietary niacin on microbial taxa and potential functional capacities of rumen and cecum microbiome in experimental cattle. In overall, dietary niacin significantly changed the α- and β-diversity of rumen microbiome. At the phylum level, the α-diversity indices including observed species, Shannon, and Chao 1 were significantly decreased by dietary niacin (*p* < 0.05). However, the Simpson index were significantly increased (*p* < 0.05) (Supplementary Figure S3A). At the species level, only observed species and Chao 1 indices were significantly decreased (*p* < 0.05), and the difference in Shannon and Simpson indices did not achieve the significance level (Figure 3A). Furthermore, dietary niacin enlarged the diversity of microbial compositions between two groups and among individuals within the niacin group from the β-diversity analysis (Figure 3B, Supplementary Figure S3B).

We then identified microbial taxa in the rumen microbiome responding to the niacin treatment by LefSe analysis at the significance level of |LDA| ≥ 2.0 and *p* < 0.05. Seven phyla were identified to show significantly different abundances between niacin and control groups. Fibrobacteres and Euryarchaeota were significantly enriched in the control group, while Firmicutes, Proteobacteria, Chloroflexi, Thermotogae, and Synergistetes had a higher abundance in the niacin group (Figure 3C). At the genus level, we identified 22 microbial genera showing different abundances between niacin and control groups, including *Fibrobacter*, *Schaedlerella*, and *Methanocorpusculum* enriched in the control group. A total of 19 genera including *Selenomonas*, *Clostridium*, *Butyrivibrio*, *Roseburia*, *Succinivibrio*, and *Ligilactobacillus* had a higher abundance in the niacin group (Supplementary Figure S3C). At the species level, we found 18 and 11 species that were enriched in the control and niacin group, respectively (Figure 3D; Supplementary Table S9). Particularly, *Selenomonas ruminantium*, *Butyrivibrio fibrisolvens*, *Ligilactobacillus ruminis*, and *Succinivibrio dextrinosolvens* were enriched in the niacin group. However, two *Prevotella* spp. (*Prevotella ruminicola* showed the most significance), two *Fibrobacter* spp. and two *Ruminococcus* spp. were enriched in the control group (Supplementary Table S9).

**Figure 4:**
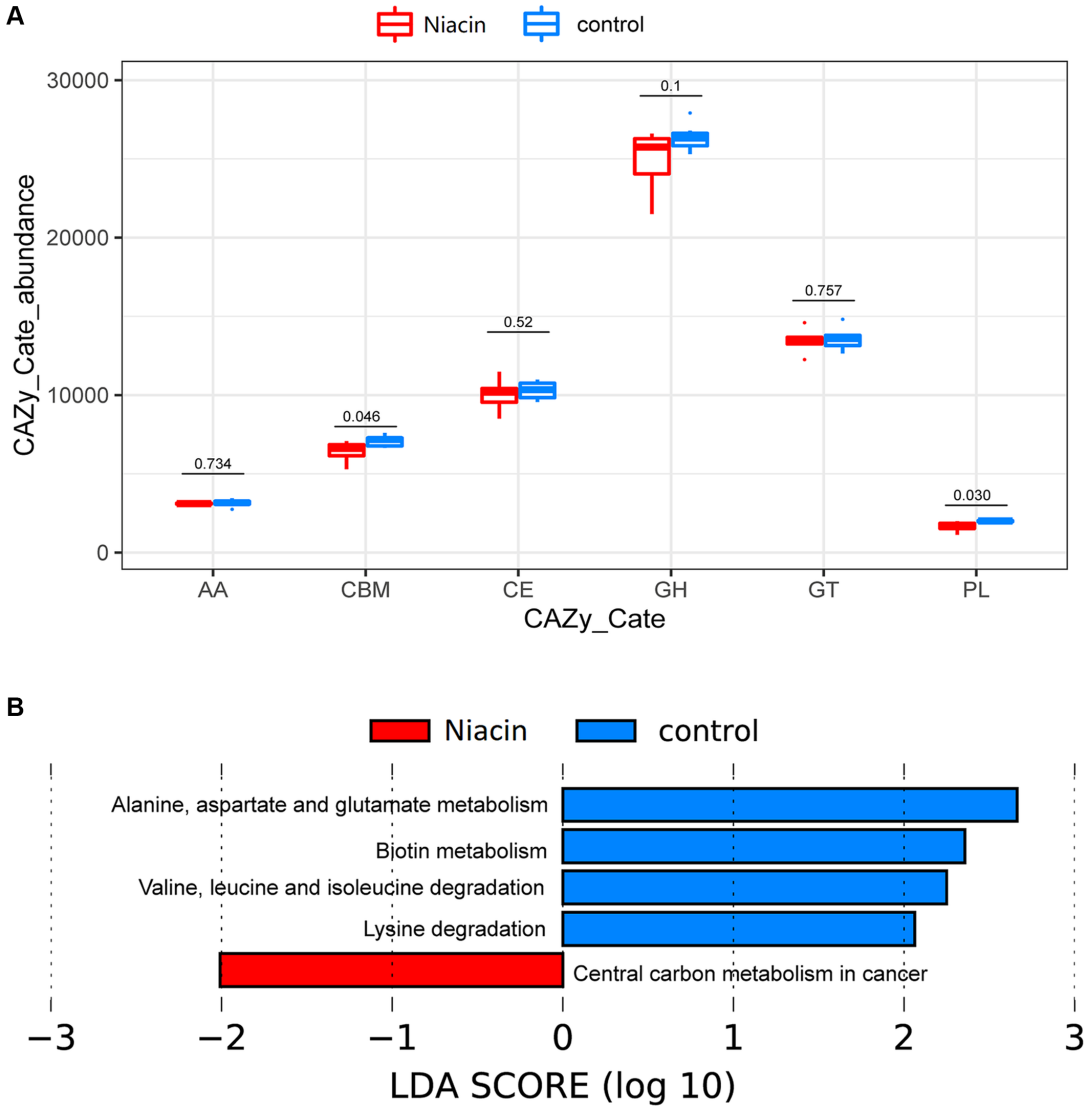
The effect of dietary niacin on functional capacities of rumen microbiome. **(A)** Comparison of the abundances of CAZymes between control and Niacin groups. The abundances of Carbohydrate Binding Modules (CBMs) and Polysaccharide Lyases (PLs) were decreased by dietary niacin. **(B)** The KEGG pathways affected by dietary niacin. Differential KEGG pathways between niacin and control groups were identified by LefSe analysis at the significance threshold of LDA ≥ 2 and *p* < 0.05.

We further identified the functional capacities of rumen microbiome affected by dietary niacin. For six CAZyme categories identified in this study, only CBM and PL were significantly affected by dietary niacin. Their abundances were significantly decreased by dietary niacin (*p* < 0.05, Figure 4A). For KEGG pathways, a total of five KEGG pathways showed different abundances between niacin and control groups. Alanine, aspartate and glutamate metabolism, Biotin metabolism, Valine, leucine and isoleucine degradation, and Lysine degradation were enriched in the control group, while the pathway Central carbon metabolism in cancer was enriched in the niacin group (LDA ≥ 2.0 and *p* < 0.05) (Figure 4B). Interestingly, the pathway of Carbohydrate digestion and absorption had a higher abundance in the niacin group although this enrichment did not achieve significance level (LDA = 1.770, *p* = 0.035).

We did not observe the significant effect of dietary niacin on the microbial composition of cecum microbiome from either the α- or β-diversity analysis (Supplementary Figure S4). At the phylum level, only Thermotogae and Apicomplexa showed different abundances between niacin and control groups. Both phyla had extremely low abundance in tested samples, and Apicomplexa was only detected in the niacin group (Supplementary Figures S5A,B). At the genus and species level, only three genera showed the tendency of differential abundances between the two groups, including *Methanocorpusculum* enriched in the control group, and *cellulosllticum* and *Vibrio* having higher abundance in the niacin group. However, it did not achieve significance level (Supplementary Figure S5C). Two species were enriched in the control group above the significance level (LDA > 2 and *p* < 0.05), but we did not identify any species enriched in the niacin group (Supplementary Figure S5D). The similar conditions were also observed in the functional capacity profiles of cecum microbiome. Dietary niacin showed no effect on the CAZymes (Supplementary Figure S6A). However, the KEGG pathway of Folate biosynthesis were enriched in the control group, whereas the pathways Sulfur relay system and Thiamine metabolism showed the enrichment in the niacin group (LDA ≥ 2.0 and *p* < 0.05) (Supplementary Figure S6B).

### 3.5 Association of the niacin-regulated microbial species and functional capacities of rumen microbiome with fat deposition traits in experimental cattle

**Figure 5:**
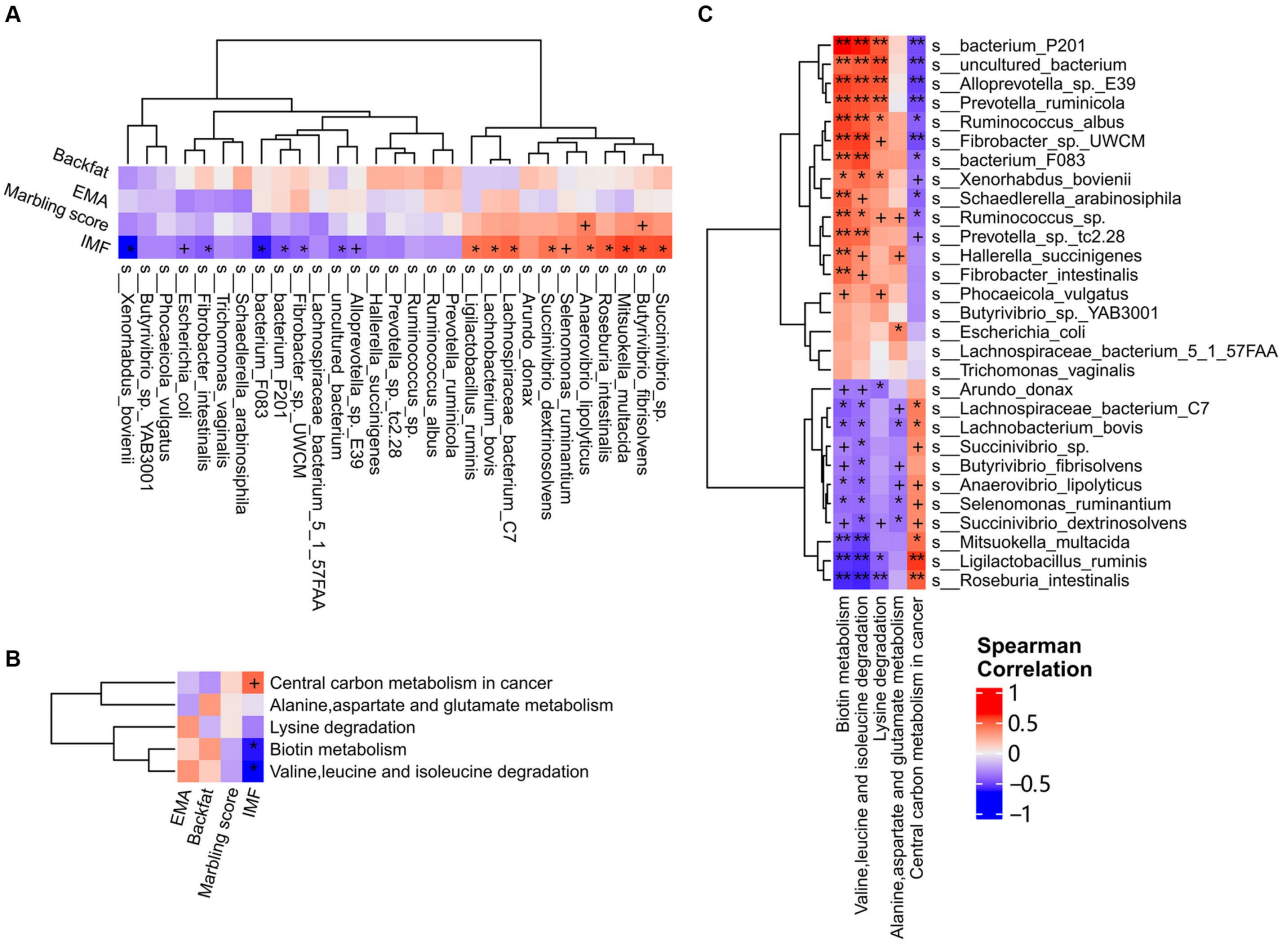
The correlations of the niacin-regulated species and functional pathways of rumen microbiome with fat deposition traits. **(A)** Correlations between niacin-regulated microbial species and fat deposition traits. Fat deposition traits including backfat thickness, eye muscle area, marbling score, and intramuscular fat content (IMF) were well phenotyped and used in the analysis. **(B)** Correlations between niacin-regulated functional capacities and fat deposition traits. **(C)** Correlations between niacin-regulated microbial species and functional capacities of rumen microbiome. Spearman correlation analysis was performed and the coefficients were calculated. Red represents positive correlations and blue indicates negative correlations. The stars in the grid represent the significance threshold: +, *p* < 0.1; *, FDR < 0.2 and p < 0.05; and **, FDR < 0.05 and *p* < 0.01.

In our previous study, we found that dietary niacin significantly favored intramuscular fat deposition and lipid metabolism in castrated finishing steers used in this study (Yang et al., 2016). To confirm the hypothesis that dietary niacin influenced fat deposition in finishing steers by regulating the microbial composition and functional capacities of rumen microbiome, we performed a correlation analysis between rumen microbial species affected by dietary niacin and host fat deposition traits including IMF, backfat, marbling, and EMA, for which the phenotypical values were reported in our previous study (Yang et al., 2016). The correlation analysis was not performed in the cecum microbiome because niacin showed no significant effect on the cecum microbiome (described above). Because of the relatively small sample size, FDR < 0.2 and *p* < 0.05 was set as the significance level and *p* < 0.1 was considered as the tendency to significant correlation. A total of 15 significant associations were identified between niacin-regulated rumen microbial species and IMF (FDR < 0.2, *p* < 0.05). Among them, nine microbial species were positively associated with IMF, including *Ligilactobacillus ruminis*, *Anaerovibrio lipolyticus*, *Succinivibrio dextrinosolvens*, *Lachnobacterium bovis*, *Lachnospiraceae bacterium C7*, *Roseburia intestinalis*, *Butyrivibrio fibrisolvens*, *Succinivibrio* sp., and *Mitsuokella multacida* (*r* = 0.55 ~ 0.73, *p* = 0.043 ~ 0.0028, and FDR < 0.2) (Figure 5A). The relative abundances of all these nine species were increased by dietary niacin (Supplementary Table S9). The other six microbial species were negatively associated with IMF, including *Xenorhabdus bovienii*, *bacterium F083*, *bacterium P201*, *Fibrobacter intestinialis*, *uncultured bacterium*, and *Fibrobacter* sp. *UWCM* (*r* = −0.54 ~ −0.83, *p* = 0.048 ~ 0.00025, and FDR < 0.2) (Figure 5A). The relative abundances of these six species were decreased by dietary niacin (Supplementary Table S9). As for marbling score, only *Butyrivibrio fibrisolvens* and *Anaerovibrio lipolyticus* showed the tendency associated with marbling score (*p* < 0.1). We did not identify any microbial species associated with backfat thickness and EMA (Figure 5A).

We then evaluated the associations of niacin-regulated functional capacities of rumen microbiome with IMF, backfat, marbling, and EMA. The pathways of Biotin metabolism, and Valine, leucine and isoleucine degradation were negatively correlated with muscle IMF content (*r* = −0.63 and − 0.65, *p* = 0.017 and 0.012, and FDR < 0.2), while the pathway of Central carbon metabolism in cancer showed the tendency of positive correlation with IMF content (*r* = 0.47, *p* = 0.091). However, we did not identify any associations between niacin-regulated functional capacities of rumen microbiome and fat deposition traits (Figure 5B).

To test whether the niacin regulated-rumen microbial taxa changed the functional capacities of rumen microbiome, and finally resulted in the different phenotypic values of fat deposition traits, we further analyzed the correlations between the niacin-regulated rumen microbial species and functional capacities. As shown in Figure 5C, the microbial species up-regulated by niacin and positively associated with IMF content were negatively correlated with the pathways of Biotin metabolism, and Valine, leucine and isoleucine degradation, but positively associated with the pathway of Central carbon metabolism in cancer (*p* < 0.05 and FDR < 0.2), and vice versa. These results suggested that dietary niacin increased muscle IMF content of beef cattle through up-regulating the abundance of rumen microbial species, such as *Mitsuokella multacida*, *Ligilactobacillus ruminis*, *Anaerovibrio lipolyticus*, and *Roseburia intestinalis* that enhanced the functional capacity of Central carbon metabolism, but lowered Biotin metabolism, and Valine, leucine and isoleucine degradation in the rumen microbiome.

## 4 Discussion

In this study, we systematically described the profiles of microbial composition and potential functional capacities of rumen and cecum microbiome in castrated finishing steers by using deep metagenomic sequencing. More importantly, we identified the microbial taxa and functional pathways that were perturbed by dietary niacin, and observed the correlations of the niacin-regulated microbial species and function capacities of rumen microbiome with host IMF content. The results provided an example about the improvement of cattle production performance by regulating the rumen microbiome with feed additives.

A comprehensive understanding and manipulation of the rumen microbiome is essential for improving the efficiency of ruminant production (Huws et al., 2018). In recent years, great advances in the understanding of rumen microbial compositions and functional capacities have been achieved by metagenomic sequencing or extensive genome sequencing of cultured rumen bacteria and archaea (Seshadri et al., 2018; Stewart et al., 2019). The rumen microbiota consists of bacteria, archaea, viruses, fungi and protozoa that ferment complex carbohydrates, such as lignocellulose and cellulose, to produce VFAs. In this study, we found that bacteria occupied more than 96.0% of relative abundance in rumen microbial composition of castrated finishing steers. *Prevotella ruminicola*, *Ruminococcus flavefaciens, Butyrivibrio fibrisolvens*, and *Selenomonas ruminantium* were the predominant bacterial species. This was consistent with the results from the previous reports (Henderson et al., 2015; Stewart et al., 2019; Xie et al., 2021), in which *Prevotella*, *Butyrivibrio*, and *Ruminococcus* were the most dominant bacteria in the rumen. Compared with that of the rumen microbiome, significantly fewer studies have been found to investigate the composition of cecum microbiome in ruminants. In this study, 10 experimental cattle were collected both rumen and cecum content samples. This facilitated to the comparison of microbial compositions and functional capacities between rumen and cecum microbiome. Xie et al. (2021) reported that *Prevotella* spp. and *Fibrobacter* spp. were the predominant bacteria in the stomach region, while *Bacteroides* spp., *Clostridium* spp., and *Alistipes* spp. were enriched in the large intestine (Xie et al., 2021). Here, we specifically identified five *Fibrobacter* spp., three *Prevotella* spp. and two *Butyrivibrio* spp. in rumen samples. However, *Clostridiales bacterium* had the highest abundances (occupied an average of 75.68% in relative abundance) in the cecum. Methane, a byproduct of ruminant fermentation that causes global climate change, is released by methanogenic archaea. Methane production has been directly related to the abundance of methanogenic archaea in the rumen (Wallace et al., 2015). In this study, we identified four *Methanobrevibacter* spp. in both rumen and cecum content samples although their relative abundances were different between two locations (0.08% vs. 0.03%). These relative abundances were lower than that reported in previous study (0.71% in the stomach and 1.1% in large intestine) (Xie et al., 2021). This discrepancy can be attributed to the variations in sample sources and treatments, because seven ruminant species were included in that previous study (Xie et al., 2021), while our study only focused on the experimental cattle provided with a concentrated diet supplemented niacin. In the aspect of functional capacities between rumen and cecum microbiome, GHs, GTs, and PLs were significantly enriched in the rumen microbiome. This was consistent with the fact that the rumen is the main location degrading complex carbohydrates (plant polysaccharides) to produce VFAs (Hess et al., 2011). On the other hand, CBMs and AAs, which play an auxiliary part in plant cell-wall hydrolysis (Levasseur et al., 2013) were enriched in the cecum microbiome.

Apart from niacin in diets, niacin synthesized by microbiota in the rumen is an important source for dairy cows. But the amount synthesized seems to differ greatly (Niehoff et al., 2009), and might be influenced by the forage-to-concentrate ratio (Seck et al., 2017). Many studies revealed a positive impact of a niacin supplementation on rumen protozoa, microbial protein synthesis, and VFAs production in the rumen (Erickson et al., 1991; Niehoff et al., 2009). In this study, we observed that dietary niacin significantly altered the microbial composition of rumen microbiome although this effect was not significant in two niacin-treated cattle. This discrepancy should be caused by different genetics (half-brothers used in this study) and other unforeseen physiological factors. We did not observe any significant changes in the cecum microbiome from either the α- or β-diversity analysis. This distinct result should be owning to the insufficient amount of niacin reaching the cecum because of the degradation or absorption of niacin in the small intestine (Niehoff et al., 2009). Apparent absorption of niacin in the duodenum was not influenced by the type of feed and accounted for 67% (Niehoff et al., 2009), 79% and 84% (Santschi et al., 2005).

Niacin significantly increased the relative abundances of several bacterial species involved in carbohydrate metabolism. *Selenomonas ruminantium* which showed the most significant difference in relative abundance between the niacin and control groups, is a nonfibrolytic bacterium that may interact with fibrolytic bacteria (Koike et al., 2003). It improves fiber digestion when co-cultured with *Ruminococcus flavefaciens* by the conversion of succinate into propionate (Chen and Weimer, 2001). The abundance of *Ligilactobacillus ruminis* and *Lachnobacterium bovis* which produce lactic acid (Whitford et al., 2001) were also increased by niacin. Interestingly, *Selenomonas ruminantium* can also ferment soluble sugars (e.g., glycerol) and lactic acid, and produce ammonia from protein hydrolysates (Takatsuka et al., 1999). This was consistent with the previous reports that niacin can avoid lactate accumulation in rumen wall (Kristensen and Harmon, 2004; Luo et al., 2017). Niacin also led to an increase in *Butyrivibrio fibrisolvens* and *Roseburia intestinalis*, which play an important role in the ruminal fermentation of polysaccharides to produce butyrate (Tamanai-Shacoori et al., 2017; Rodriguez Hernaez et al., 2018). These results were consistent with the observation that the KEGG pathways of Carbohydrate digestion and absorption, and Central carbon metabolism in cancer were enriched in niacin group, suggesting the improvement of Carbohydrate metabolism and absorption by niacin. Niacin also increased the abundances of bacterial species involved in lipid hydrolysis and utilization of nutrients. *Anaerovibrio lipolyticus* has been recognized as one of the major species involved in lipid hydrolysis in ruminant animals. Ruminal lipase activity in animals receiving mainly concentrate feeds is thought to be accomplished mainly by *Anaerovibrio lipolyticus* (Prive et al., 2013). In this study, experimental cattle were fed with 90% of concentrate feeds (Yang et al., 2016). *Mitsuokella multacida* can produce phytase activity which reduce the need to supplement diets with additional phosphates in monogastric animals (Kalmokoff et al., 2009). Niacin significantly increased the abundance of two *Succinivibrio* species. Some *Succinivibrio* strains, such as *Succinivibrio dextrinosolvens*, have been shown to possess all the necessary enzymes for degradation and assimilation of nitrogen-containing compound (Hailemariam et al., 2020). Interestingly, as mentioned above, *Selenomonas ruminantium* produce ammonia from protein hydrolysates (Takatsuka et al., 1999), so the increased abundances of these two species by dietary niacin should improve the utilization of dietary protein. *Succinivibrio dextrinosolvens* has been reported to be associated with high feed efficiency (Hailemariam et al., 2020). However, it was strange that KEGG pathways Alanine, aspartate and glutamate metabolism, Valine, leucine and isoleucine degradation, and Lysine degradation were enriched in the control group. It was not difficult to explain this because the abundance of *Prevotella ruminicola* which had the highest relative abundance in both niacin (29.27% in average) and control (21.85%) groups and plays a significant role in the metabolism of proteins and peptides in the rumen (Wallace et al., 1997) was significantly decreased by dietary niacin (enriched in the control group). Furthermore, different from the improvement of dietary protein hydrolysates by niacin-upregulated microbes, previous study also indicated that niacin could increase microbial protein synthesis although inconsistent reactions to supplemental niacin has been reported (Erickson et al., 1991; Niehoff et al., 2009). This should be another explanation for the decreased abundance of functional pathways related amino acid metabolism and degradation in the niacin group. The relative abundances of several bacterial species including two *Fibrobacter* spp., two *Ruminococcus* spp., and *Butyrivibrio_sp_YAB3001* that are involved in fiber digestion to produce VFAs (Xie et al., 2022) were also decreased by dietary niacin. Correspondingly, the abundances of the CAZymes CBM and PL were also significantly decreased by supplemental niacin. This result suggested that dietary niacin significantly decreased the abundances of several most common fiber-degrading and VFAs-producing bacteria in the rumen, such as *Fibrobacter* spp., and *Ruminococcus* spp., but increased the abundance of several butyrate-producing bacteria, such as *Butyrivibrio fibrisolvens* and *Roseburia intestinalis* in castrated finishing steers fed with 1,000 mg/kg niacin. The abundances of two pathogenic bacteria *Escherichia coli* and *Xenorhabdus bovienii* (Murfin et al., 2015) were down-regulated by dietary niacin, suggesting dietary niacin may have a beneficial effect on the health of experimental cattle.

All four archaeal species identified in this study are the members of *Methanobrevibacter* belonging to the methanogens in Euryarchaeota. According to meta-analysis of global data, 63.2% of rumen methanogens belong to the *Methanobrevibacter* (Janssen and Kirs, 2008). Interestingly, the abundances of Euryarchaeota and *Methanocorpusculum* in the rumen were significantly decreased by dietary niacin. Previous study has revealed that niacin possesses good binding affinity against Methyl co-enzyme M reductase (the target protein for methanogenesis) and should be treated as a potential inhibitor of methylcoenzyme M reductase (Dinakarkumar et al., 2021). Methane is generated in the foregut of all ruminant animals by the microbes present. Dietary manipulation has been regarded as the most effective and convenient way to increase nitrogen utilization efficiency and reduce methane emissions (and in turn energy loss in the animal). This result suggested that dietary niacin should be considered as an effective way to reduce methane emissions and energy loss.

Overall, dietary niacin up-regulated the abundances of bacterial species related to the fermentation of soluble sugars and lactic acid, the production of lactic acid and butyrate, lipid hydrolysis, and degradation and assimilation of nitrogen-containing compounds, but down-regulated the abundances of bacterial species involved in fiber digestion to produce VFAs and methane emissions. The results implied that niacin may improve nutrient (Carbohydrate, lipid and nitrogen-containing compounds) digestion and absorption, and reduce energy loss by regulating rumen bacteria. Niacin should also improve host health by down-regulated the abundances of several pathogens.

In our previous study, dietary niacin increased IMF along with no effect on backfat thickness (Yang et al., 2016). Concordantly, in this study, the correlation analysis between niacin-regulated rumen bacterial species and phenotypic values of fat deposition traits only identified the significant associations between niacin-regulated rumen bacterial species and IMF. Combining the discussion about the effect of niacin on rumen bacteria (see above), we speculated that niacin increased muscle IMF by improving nutrient (Carbohydrate, lipid, and nitrogen-containing compounds) digestion and absorption (Central carbon metabolism), and reducing energy loss, Lysine degradation, Valine, leucine and isoleucine degradation, and Biotin metabolism. Interestingly, the increased level of Valine, leucine and isoleucine has been reported to be associated with increased fat deposition (Chen et al., 2021).

In summary, in this study, we systematically exhibited and compared the profiles of microbial compositions and functional capacities of rumen and cecum microbiome, and constructed MAGs. Especially, we found significant effects of dietary niacin on rumen microbiome, but not on cecum microbiome. We further identified the rumen bacterial species regulated by dietary niacin. Finally, we suggested the possible mechanism of niacin increasing muscle IMF by regulating rumen microbiome. This study provides the knowledge and suggestions that dietary manipulation, such as niacin supplementation, should be regarded as the effective and convenient way to improve meat quality (IMF) and production performance, e.g., feed efficiency. It will help the beef cattle industry.

## Data availability statement

The datasets presented in this study can be found in online repositories. The names of the repository/repositories and accession number(s) can be found at: https://ngdc.cncb.ac.cn/gsub/submit/gsa/list, CRA012971.

## Ethics statement

All processes related to experimental animals were conducted in accordance with the guidelines established by the Ministry of Agriculture and rural affair of China. The Animal Care and Use Committee (ACUC) of Jiangxi Agricultural University approved this study (No. JXAU2011-006). The study was conducted in accordance with the local legislation and institutional requirements.

## Author contributions

ZY: Conceptualization, Data curation, Formal analysis, Funding acquisition, Investigation, Methodology, Validation, Visualization, Writing – original draft, Writing – review & editing. XC: Formal analysis, Writing – review & editing. MY: Investigation, Resources, Writing – review & editing. RJ: Investigation, Writing – review & editing. LB: Investigation, Writing – review & editing. XZ: Investigation, Writing – review & editing. KP: Investigation, Methodology, Writing – review & editing. BC: Investigation, Writing – review & editing. MQ: Supervision, Writing – review & editing.
