# Supplementary material for: Metagenomic sequencing identified microbial species in the rumen and cecum microbiome responsible for niacin treatment and related to intramuscular fat content in finishing cattle
Source: Front Microbiol. 2024 Mar 11;15:1334068. doi: 10.3389/fmicb.2024.1334068 (PMC10961399; doi:10.3389/fmicb.2024.1334068)
Supplement: Supplementary file 2 [file Table_1.DOCX]

**Supplementary information**

**Metagenomic sequencing identified microbial species in the rumen and cecum microbiome responsible for the treatment of Niacin and related to intramuscular fat content in finishing cattle**

Zhuqing Yang^1*^，Mingjin Yu^1^，Xiao Chen^1^，Ruixue Jing^1^，Linbin Bao^2^，Xianghui Zhao^1, 2^，Ke Pan^1,2^，Mingren Qu^1,^ ^2*^

^1^College of Animal Science and Technology, Jiangxi Agricultural University, Nanchang 330045, China.

^2^Jiangxi Provincial Key Laboratory for Animal Nutrition/Engineering Research Center of Feed Development, Jiangxi Agricultural University, Nanchang, 330045, China.

Zhuqing Yang，[yangzhuqingjxau@hotmail.com](mailto:yangzhuqingjxau@hotmail.com)

Mingjin Yu，[mingjin2000@163.com](mailto:mingjin2000@163.com)

Xiao Chen，chen15628699251@163.com

Ruixue Jing，jrx2686736602@163.com

Linbin Bao，[bao.200802@163.com](mailto:bao.200802@163.com)

Xianghui Zhao，[zhaoxh001@163.com](mailto:zhaoxh001@163.com)

Ke Pan, [panke6789@tom.com](mailto:panke6789@tom.com)

Mingren Qu，[qumingren@126.com](mailto:qumingren@126.com)

**^*^Corresponding Author: Zhuqing Yang**

Tel: +0086-791-83813503, Fax: +0086-791-83813504, E-mail: yangzhuqingjxau@hotmail.com

**Mingren Qu**

Tel: +0086-791-83813503, Fax: +0086-791-83813504, E-mail: [qumingren@126.com](mailto:qumingren@126.com)

**Runing title:** Niacin-regulated rumen microbial species were associated with IMF


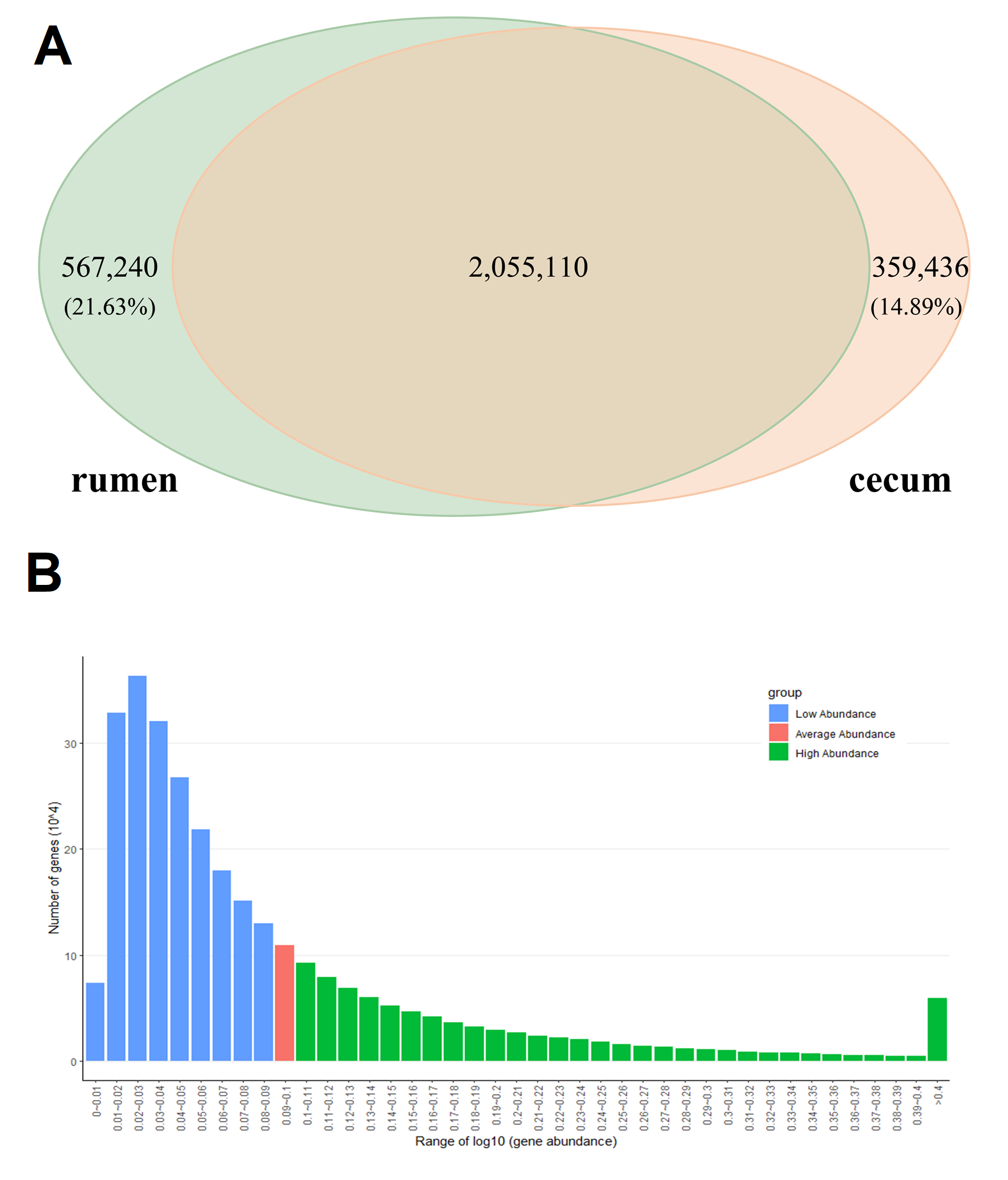


**Figure S1. Microbial genes identified in rumen and cecum content samples, and their abundances.**

A. Numbers (percentages) of microbial genes identified in both rumen and cecum content samples, or specifically identified in each of two types of samples. B. The distribution of the abundances of 2,981,786 non-redundant genes in tested samples. The *x*-axis indicates the ranges of log _10_ (gene abundance), and the y-axis shows the gene number in each abundance range.


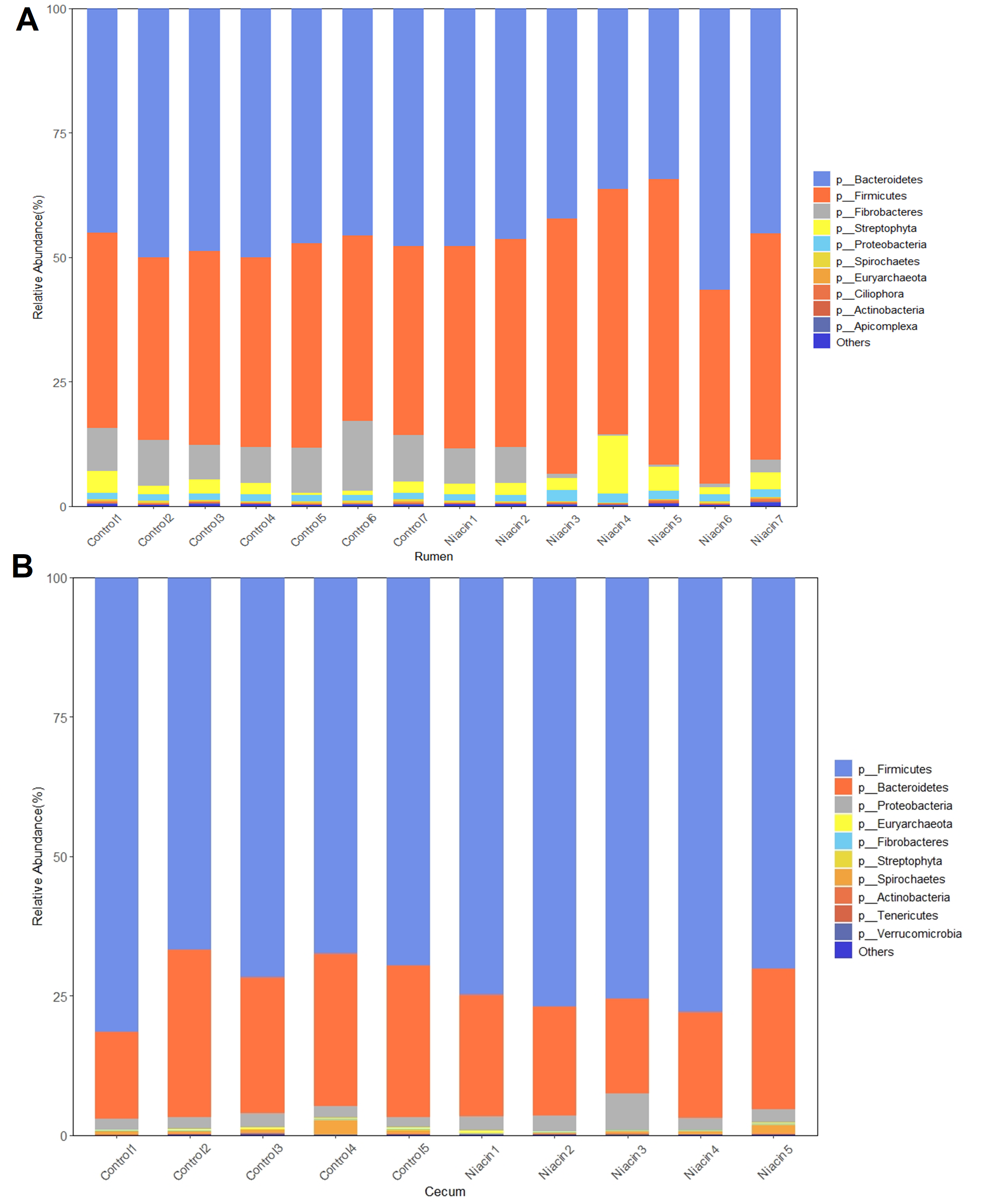


**Figure S2. The histograms showing the microbial compositions of rumen and cecum content samples at the phylum level.**

A. rumen samples; B. cecum content samples


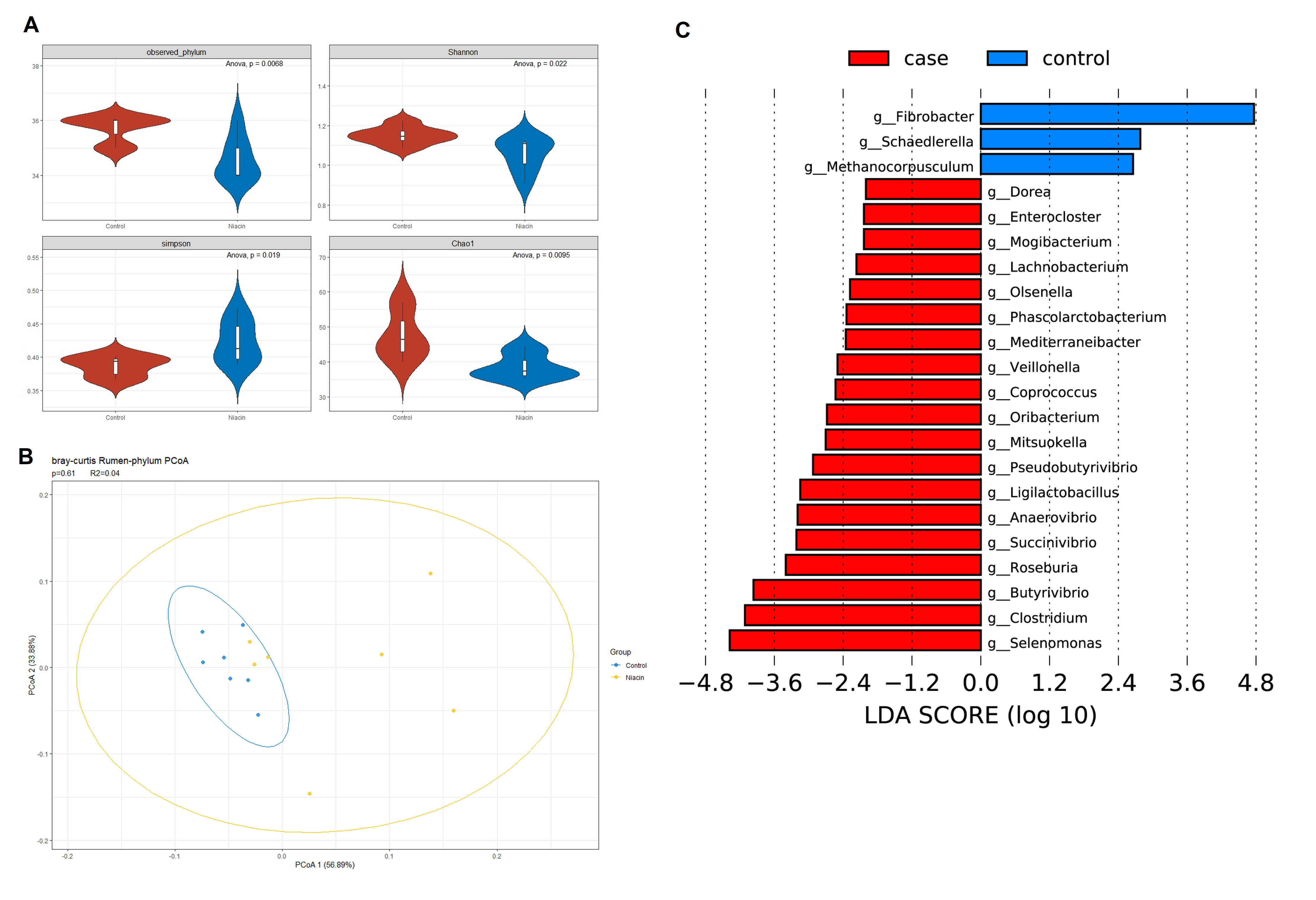


**Figure S3. The effect of dietary Niacin (NA) on rumen microbial composition.**

A. The effect of dietary NA on the α-diversity of rumen microbiome at the phylum level. The indices of observed species, Shannon, Simpson, and Chao 1 were compared between control and Niacin groups using ANOVA analysis. *P* < 0.05 was treated as the significance threshold. B. The effect of dietary NA on the β-diversity of rumen microbiome at the phylum level. The β-diversity was evaluated based on the bray-curtis distance. C. Differential microbial genera identified by LefSe analysis between Niacin and control groups at the significance threshold of LDA ≥ 2 and *P* < 0.05.


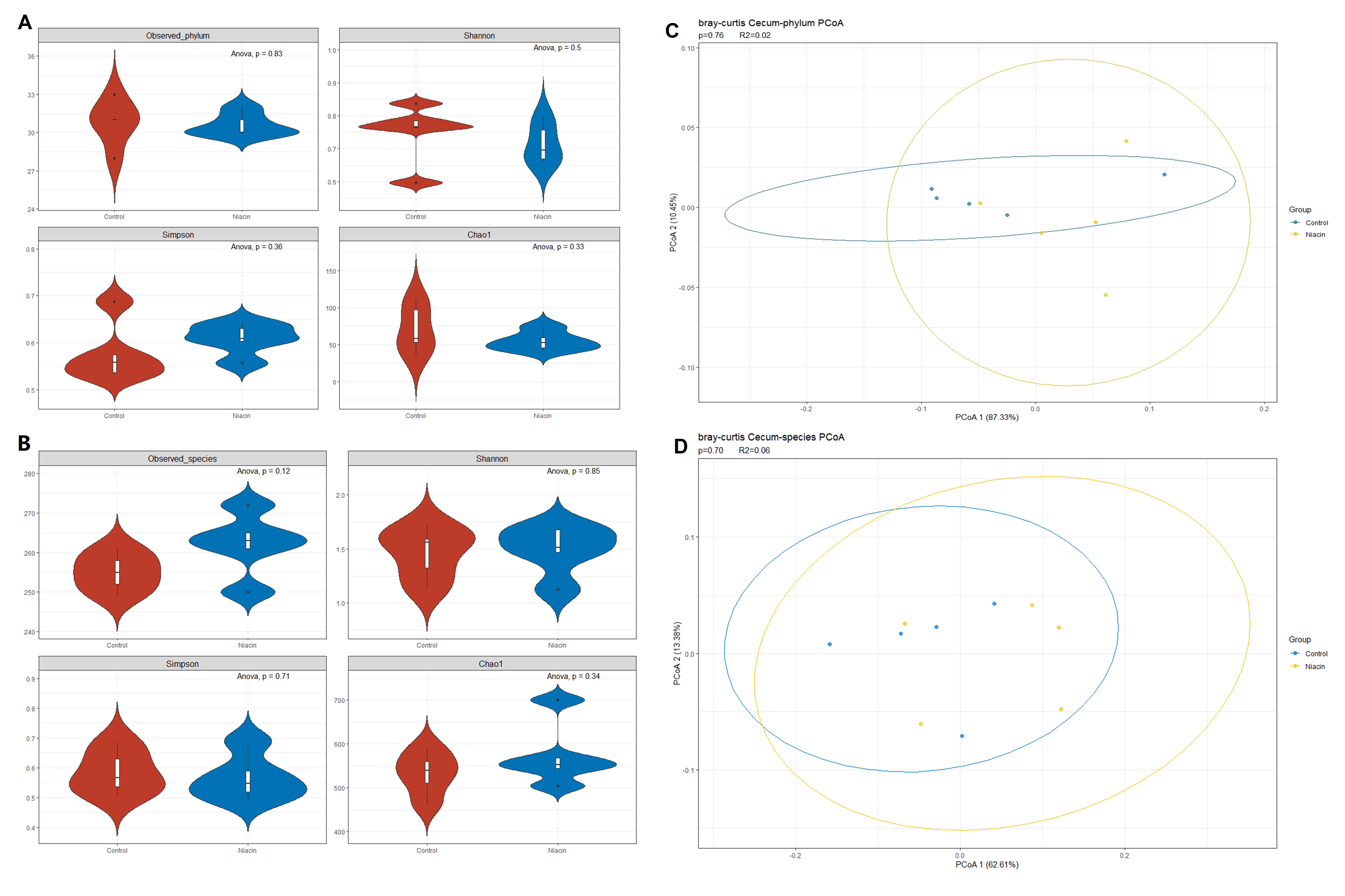


**Figure S4. The effect of dietary Niacin (NA) on the microbial composition of cecum content samples.**

A and B. The effect of dietary NA on the α-diversity of cecum microbiome at the phylum (A) and species (B) level. The indices of observed species, Shannon, Simpson, and Chao 1 were compared between control and Niacin groups using ANOVA analysis. *P* < 0.05 was treated as the significance threshold. **C and D**. The effect of dietary NA on the β-diversity of cecum microbiome at the phylum (C) and genus (D) level. The β-diversity was evaluated based on the bray-curtis distance.


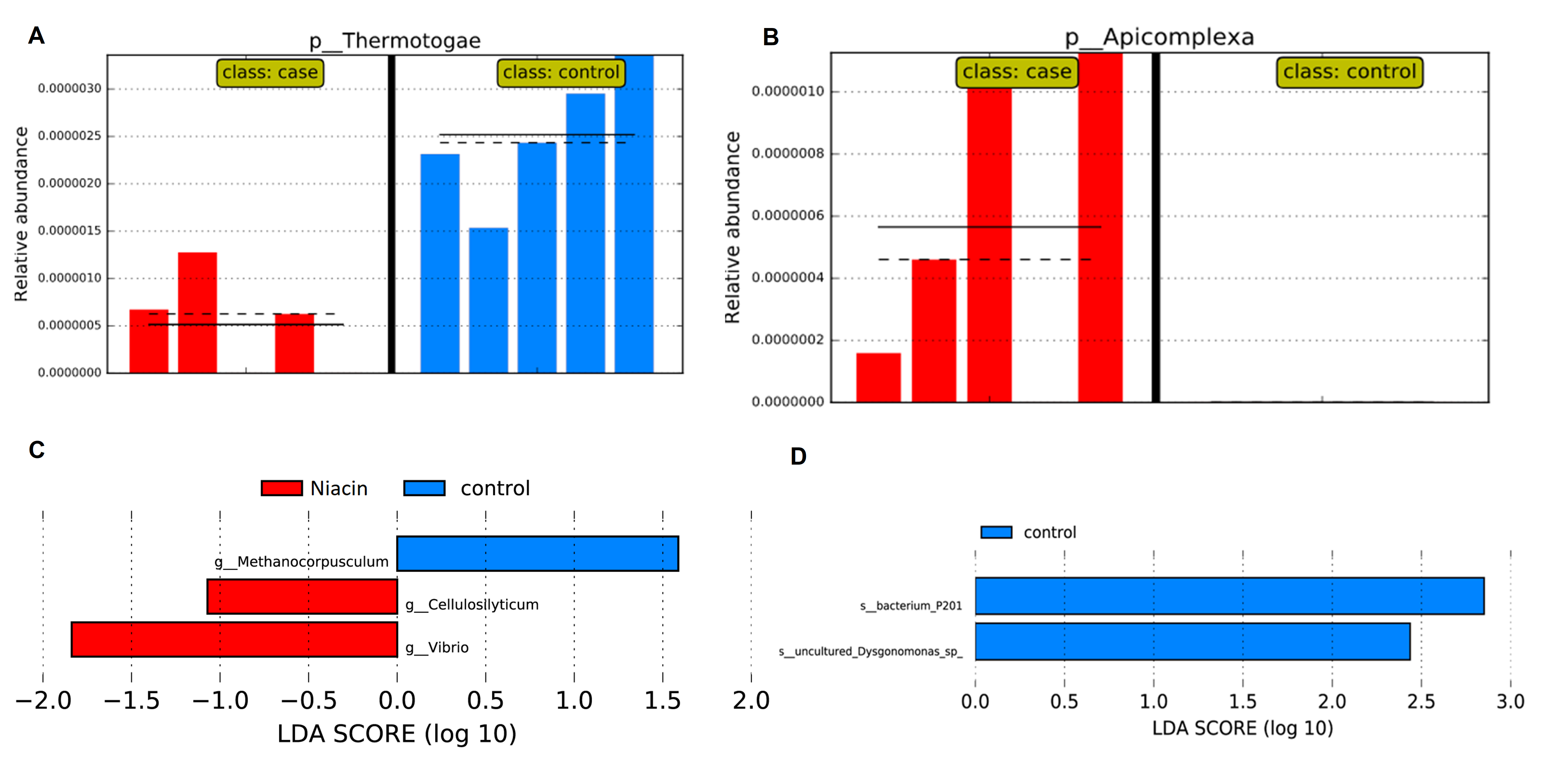


**Figure S5. Microbial taxa affected by dietary niacin**

A. Thermotogae showing the enrichment in the control group. B. Apicomplexa only identified in the niacin group. C. Microbial genera showing the tendency of differential enrichments between Niacin and control groups by LefSe analysis. D. Two species enriched in the control group by LefSe analysis.


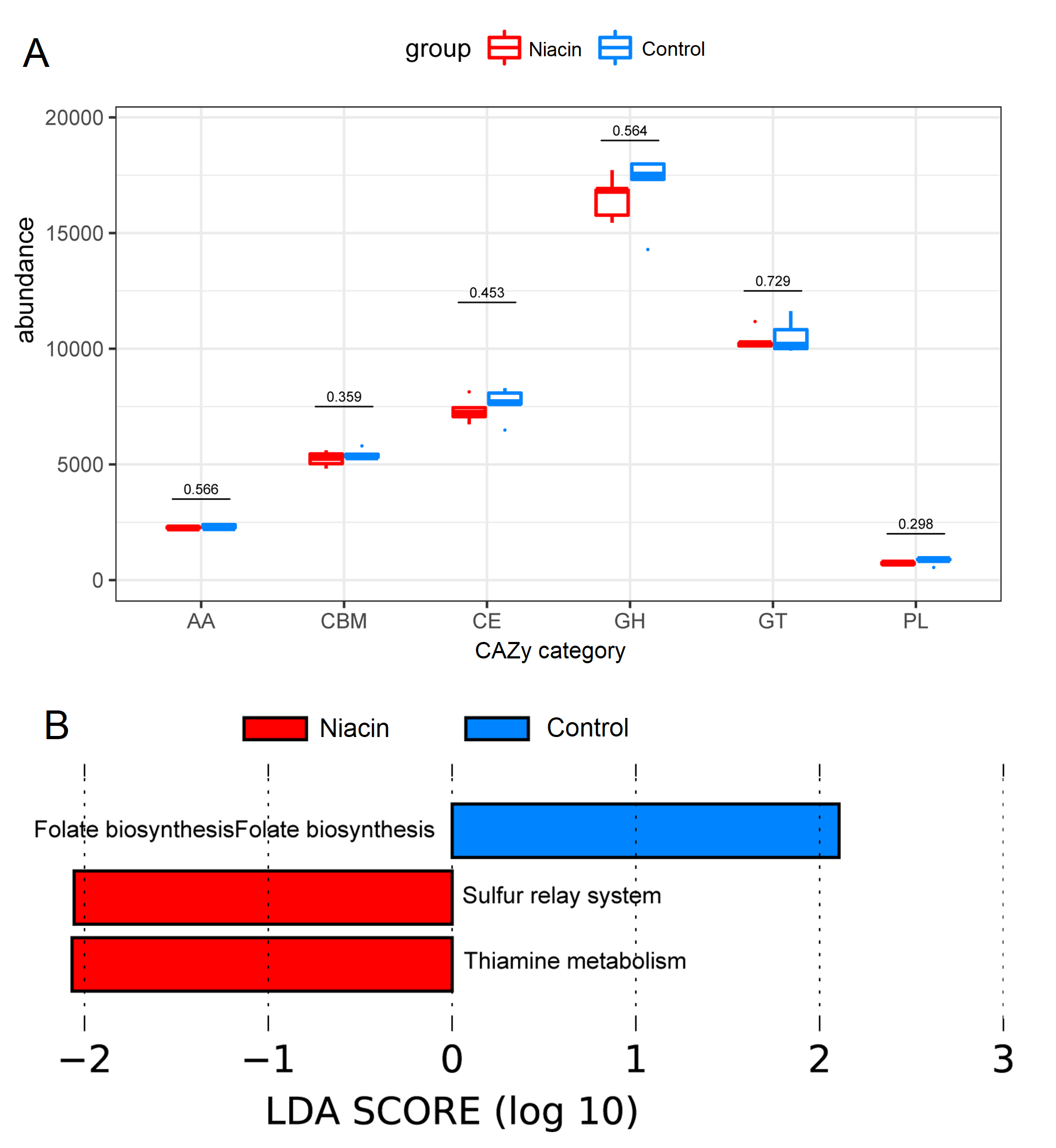


**Figure S6. The effect of dietary Niacin (NA) on functional capacities of cecum microbiome.**

A. The effect of dietary NA on CAZymes. B. The KEGG pathways affected by dietary NA. Differential KEGG pathways between Niacin and control groups were identified by LefSe analysis at the significance threshold of LDA ≥ 2 and *P* < 0.05.
